# Trends and changes in prescription opioid analgesic dispensing in Canada 2005–2012: an update with a focus on recent interventions

**DOI:** 10.1186/1472-6963-14-90

**Published:** 2014-02-26

**Authors:** Benedikt Fischer, Wayne Jones, Jürgen Rehm

**Affiliations:** 1 Centre for Applied Research in Mental Health and Addiction (CARMHA), Faculty of Health Sciences, Simon Fraser University, Vancouver, Canada; 2 Social and Epidemiological Research, Centre for Addiction and Mental Health (CAMH), Toronto, Canada; 3 Department of Psychiatry, University of Toronto, Toronto, Canada; 4 Dalla Lana School of Population Health, University of Toronto, Toronto, Canada; 5 Technische Universität, Dresden, Germany

**Keywords:** Prescription opioids, Oxycodone, Health policy, Canada, Population health

## Abstract

**Background:**

Prescription opioid analgesic (POA) utilization has steeply increased globally, yet is far higher in established market economies than elsewhere. Canada features the world’s second-highest POA consumption rates. Following increases in POA-related harm, several POA control interventions have been implemented since 2010.

**Methods:**

We examined trends and patterns in POA dispensing in Canada by province for 2005–2012, including a focus on the potential effects of interventions. Data on annual dispensing of individual POA formulations – categorized into ‘weak opioids’ and ‘strong opioids’ – from a representative sub-sample of 5,700 retail pharmacies across Canada (from IMS Brogan’s Compuscript) were converted into Defined Daily Doses (DDD), and examined intra- and inter-provincially as well as for Canada (total).

**Results:**

Total POA dispensing – driven by strong opioids – increased across Canada until 2011; four provinces indicated decreases in strong opioid dispensing; seven provinces indicated decreases specifically in oxycodone dispensing, 2011–2012. The dispensing ratio weak/strong opioids decreased substantively. Major inter-provincial differences in POA dispensing levels and qualitative patterns of POA formulations dispensed persisted. Previous increasing trends in POA dispensing were reversed in select provinces 2011–2012, coinciding with POA-related interventions.

**Conclusions:**

Further examinations regarding the sustained nature, drivers and consequences of the recent trend changes in POA dispensing – including possible ‘substitution effects’ for oxycodone reductions – are needed.

## Background

The global utilization of prescription opioid analgesics (POAs) – principal medications for pain care – has sharply increased in recent years. For example, the production of morphine doubled 1992–2011, and the production of oxycodone tripled 2002–2011 [1]. However, the global distribution of POA utilization is highly inequitable [2,3]. About 90% of all POAs are consumed in established market economies (EMEs), and >80% of the world’s population – mainly in low or middle income countries – have no or inadequate access to POAs, even though they have most of the world’s cancer and HIV patients [3]. Even within EMEs, North America – i.e., the United States [51,081 Defined Daily Doses per 1,000,000 population (DDD) in 2009–11], and Canada [28,731 DDD] – features far higher rates of POA utilization than any other global region [1,4]. For comparison, North America’s POA consumption rates are more than double those of the European Union or the Australia/New Zealand regions, and hundreds of times those of China or India. The disproportionately high POA use levels in North America have been explained by a multiplicity of drivers, including a strong focus on pharmacotherapeutic interventions, laxer regulatory frameworks, aggressive pharmaceutical advertising and commodification of health care, together contributing to an environment of medical care where pharmaceutical interventions are commonly privileged by providers and desired by patients over other interventions [5-8].

The high and rising POA use rates specifically in Canada – similar to the US – have been paralleled by substantive levels of POA-related morbidity and mortality [4]. In Ontario, some 6% of adults, and 15% of high-school students reported non-medical POA use in 2010/2011; POA-related substance use treatment admissions more than doubled, and POA-related overdose deaths more than tripled since 2002 [4,9-13]. Levels of POA availability have been shown to be strongly correlated with levels of POA-related morbidity and mortality, hence constituting a principal driver for POA-related harm on a population level [10,14-17].

A recent examination of POA dispensing – an imperfect yet measurable and best available indicator of POA consumption on a population level - in Canada in 2005–2010 found that [18]: 1) Most provinces featured increases in overall POA use levels; 2) increases were predominantly driven by increases in strong (versus weak, i.e. non-codeine versus codeine-based POA formulations) POA use; 3) there were considerable quantitative (i.e., overall POA use levels) and qualitative (i.e., individual POA types used) inter-provincial differences; in most provinces, oxycodone (e.g., Oxycontin®) constituted the most commonly consumed single ‘strong opioid’ formulation, and most strongly contributed to POA use increases.

Beginning in 2010, rising POA use and harm levels in Canada began to receive increasing attention from key policy, professional and mass media entities; many of these focused on Oxycontin®, associated with a large proportion of POA-related harm [4,11,19]. For example, the Ontario College of Physicians and Surgeons’ report ‘Avoiding Abuse, Achieving a Balance: Tackling the Opioid Public Health Crisis’ (2010) presented recommendations to reduce POA misuse and diversion; a multi-disciplinary workgroup launched the ‘Canadian Guidelines for Safe and Effective Use of Opioids for Chronic Non-Cancer Pain’ (2010); a high-profile coroners’ inquest into a series of POA-related deaths in Ontario (2011) made recommendations for improved POA controls; and the government of Ontario’s ‘Narcotics Expert Advisory Panel’ (2011) conveyed recommendations towards reduced POA-related misuse and harm [20-23]. These included, as a key policy measure, the delisting of Oxycontin® (together with its successor product, Oxy-Neo®), the principally common oxycodone formulation, from Ontario’s provincial drug formulary as of March 2012; this measure was replicated by the majority of other but not all provinces (e.g., Alberta) [23-25]. ‘Delisting’ meant that provincially funded drug plans (e.g., covering individuals receiving public welfare, disability or seniors’ benefits; the Ontario Drug Benefit (ODB) plan covers about 20% of the Ontario population) would not pay for these oxycodone formulations any longer, although these may still be prescribed to patients with private drug plans and/or paying out of pocket. In addition, key media outlets ran numerous prominent feature reports on increases in POA utilization, harm and policy challenges in Canada in this period [26-30].

The objective of this study was to provide an update of POA dispensing trends and patterns, by province, for the period 2005–2012, with specific consideration of recent POA-focused interventions in Canada.

## Methods

The present analyses are based on data for dispensing of POAs from retail pharmacies in Canada (meaning here: the total of the ten provinces, not including territories) for the period January 2005 to December 2012, obtained from the IMS Brogan’s (IMSB’s) Canadian CompuScript Audit [31]. It is estimated that about 80% – i.e., the large majority – of the total of POAs are dispensed by way of retail pharmacies (other main routes include hospital- or emergency care-based dispensing which are not captured in these data) [18]. The IMSB’s CompuScript panel is drawn from a representative and stratified base sample of 5,700 retail pharmacies (representing about two-thirds of the total of retail pharmacies) across Canada, from which a continuously refreshed sub-sample of 65% are providing pharmaceutical dispensing data on a monthly basis [31,32]. Following quality control checks, IMSB projects the sample data to the universe of pharmacies by province; the sampling error is estimated to be 3%–5%. Given the sampling approach described, the level of representativeness of IMSB data for the actual total of POAs dispensed by retail pharmacies in Canada is considered high.

Monthly dispensing data on all POA types were aggregated to the yearly data. Methadone was excluded from the analyses since it is primarily used for addiction (i.e., opioid maintenance) treatment, and only in less common instances for pain treatment; this results in irregular dispensing patterns, as a substantial proportion of methadone is dispensed not by retail pharmacies, and therefore non-comparable data with other POs for the purposes of the present study. Data on the different POA types dispensed in Canada during the study period were converted to DDD values – the assumed average maintenance dose per day for a drug used for its main indication for an average adult – according to the World Health Organization’s (WHO) Anatomical Therapeutic Chemical (ATC) classification and DDD measurement methodology [33,34]. Furthermore, based on the WHO’s pain ladder, codeine and its combination products were defined as ‘weak opioids', whereas hydrocodone, hydromorphone, oxycodone, fentanyl, meperidine, and morphine formulations were defined as ‘strong opioids’ for the purpose of combination analysis [35]. On this basis, and applying corresponding provincial population estimates from Statistics Canada [36], the annual dispensing rates for each PO, as well as for ‘weak POs’ and ‘strong POs’ were calculated for each province, as well as the Canada total, as the number of DDDs per 1000 population/day, and compared inter-jurisdictionally and over-time. As an additional indicator, we calculated the annual provincial ratios of dispensing of weak POs/strong POs for the years 2005 and 2012 each. Changes in the ratios (10 pairs) were tested for significance by McNemar test.

## Results

Throughout each year of the study period, Alberta featured the highest, and Quebec had the lowest total POA dispensing levels; there was a greater than 3-fold difference in annual total POA dispensing between these two provinces. In all but one province (Ontario), annual total POA dispensing (i.e., weak and strong POAs combined) increased from 2005 to 2012; four provinces (BC, AB, SK, ON; see Table 1 for list of provinces’ full names) indicated a decrease in overall POA dispensing levels in 2011–2012. While strong POA dispensing increased substantively in each province between 2005 and 2011, four provinces (BC, AB, MN, ON) indicated decreases in 2011–2012. Dispensing of oxycodone formulations – as a specific sub-group of strong opioids – increased in all provinces 2005–2011, yet decreased in seven provinces 2011–2012. Weak opioid dispensing remained overall levelled – except for Manitoba, which featured substantive increases – in most provinces 2005–2012. The ratio of weak/strong POA dispensing decreased for each province between the years 2005 and 2012; while this ratio was 1 or greater for all provinces in 2005, it was 1 or smaller for half the provinces in 2012. These changes overall approached but did not reach statistical significance (exact McNemar’s test p-value: 0.0625).

**Table 1:** Annual change rates and ratios for opioid dispensing in Canada, by province and for Canada (total), 2005–2011

	**Change rate (%) in dispensing**						**Opioid dispensing ratio**	
	**Strong opioids**		**Weak opioids**		**Oxycodone**		**Weak/Strong**	
**Province**	**2005-2011**	**2011-2012**	**2005-2011**	**2011-2012**	**2005-2011**	**2011-2012**	**2005**	**2012**
British Columbia (BC)	+48.5	-2.0	-3.8	-1.6	+135.0	-9.0	2.7	1.8
Alberta (AB)	+29.3	-3.9	-0.4	+1.7	+64.7	-9.7	2.8	2.2
Saskatchewan (SK)	+98.7	+1.2	+26.2	-3.4	+88.2	-18.1	1.5	0.9
Manitoba (MN)	+98.2	-2.9	+37.4	+3.0	+118.2	-11.5	4.2	3.1
Ontario (ON)	+39.5	-15.2	-12.3	-7.7	+75.2	-24.4	1.4	0.9
Quebec (QC)	+45.4	+2.9	-5.4	-2.2	+88.5	+0.6	1.0	0.6
New Brunswick (NB)	+39.0	+1.2	-0.7	+0.1	+56.9	-8.4	1.5	1.1
Nova Scotia (NS)	+43.5	+2.7	-4.6	-2.6	+29.1	-9.7	1.3	0.8
Prince Edward Island (PE)	+62.8	+6.0	-3.8	-3.2	+98.4	+2.1	1.8	1.0
Newfoundland (NL)	+71.8	+5.2	-1.3	+5.0	+93.3	+2.0	2.5	1.5
Canada (CA)	+43.4	-7.9	-3.3	-2.8	+80.4	-17.2	1.7	1.2

Annual change rates for ‘strong opioid’, ‘weak opioid’ and ‘oxycodone’ dispensing (2005–2011 and 2011–2012) and annual dispensing ratios weak/strong opioids (2005 and 2012), by province and Canada total.

**Figure 1:**
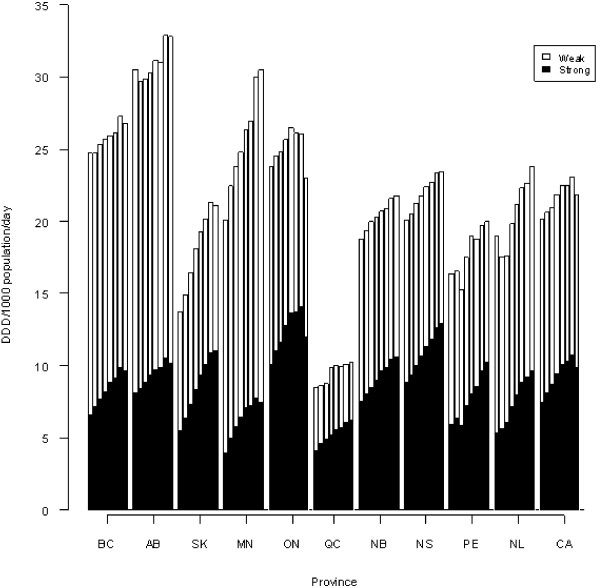
**Annual opioid dispensing (not including methadone) in Canada, 2005–2012.** Annual opioid dispensing (not including methadone), by weak opioids and strong opioids, in DDD/1000 population/day by province and Canada total, 2005–2012. Bars are chronological for years 2005–2012. For full names of provinces, see Table 1. CA represents Canada (total).

**Figure 2:**
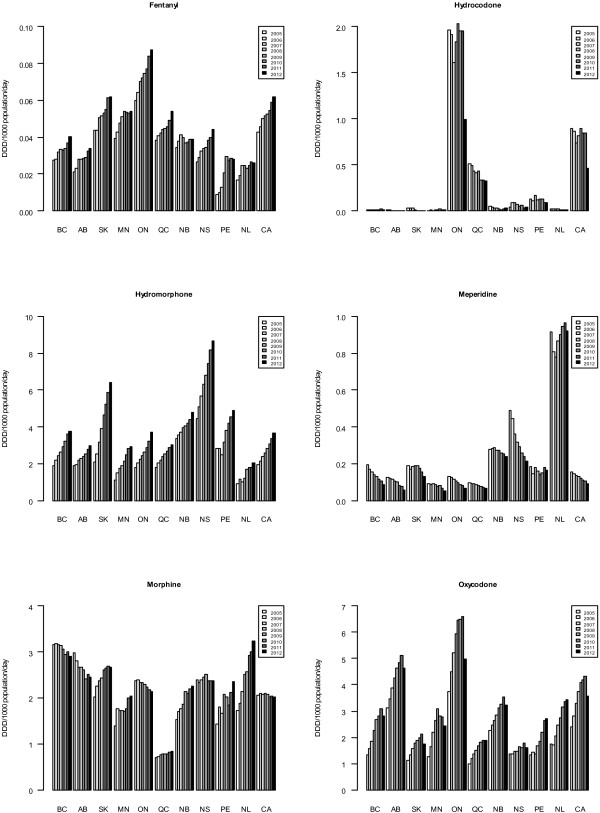
**Annual dispensing of select strong opioids in Canada, 2005–2012.** Annual dispensing of select strong opioids, in DDD per 1000 population/day, by province and Canada total, 2005–2012. Bars are chronological for years 2005–2012. For names of provinces, see Table 1. CA represents Canada (total).

Figures 1 and 2 focusing on individual (strong) POA formulations and comparing the years 2005 to 2012, fentanyl dispensing increased in all provinces, and was highest in Ontario (both years), and lowest in Prince Edward Island (2005) and Newfoundland (2012). Hydrocodone dispensing decreased in most provinces, yet was far higher in Ontario than any other province (both years), and lowest in Manitoba (2005) and Alberta (2012). Hydromorphone dispensing consistently increased in all provinces, and was highest in Nova Scotia and lowest in Newfoundland (both years, respectively). Meperidine dispensing decreased in the majority of provinces, and was highest in Newfoundland and lowest in Manitoba (both years). Morphine dispensing increased and decreased in about half of the provinces, respectively, and was highest in British Columbia (2005) and Newfoundland (2012), and lowest in Quebec (both years). Oxycodone dispensing was highest in Ontario (both years) and lowest in Quebec (2005) and Nova Scotia (2012; for over-time dispensing trends for oxycodone see above).

## Discussion

First, our analyses extend observations about key patterns and trends in POA dispensing in Canada observed in earlier examinations [18]. Concretely, POA dispensing levels substantively increased in the study period, except for notable decreases between 2011 and 2012 (see also below). The observed previous increases in POA utilization had been driven largely by increases in ‘strong opioids’ dispensing; consequently, half of the provinces (in DDDs) dispensed more ‘strong opioids’ than ‘weak opioids’ in 2012. Furthermore, considerable inter-provincial heterogeneity between the provinces regarding quantities and types of individual POs dispensed continued, including the remarkably substantive (i.e., three-fold) differences in POA dispensing levels between the highest (Alberta) and lowest (Quebec) province. It may be assumed that – similar to explanations of differences in POA utilization on inter-national levels – the inter-provincial quantitative and qualitative differences in POA utilization observed in Canada are a result of a multitude of factors, including key differences in drug regulation or formularies, monitoring and especially reimbursement schemes (all of which are set independently on provincial levels) as well as details of medical culture and practice [6,37-39]. Our observations are set in the context of Canada where utilization levels of other key psychotropic drugs (e.g., benzodiazepines, anti-depressants) have also substantively increased in recent years (e.g., 1998–2007), yet also display considerable inter-provincial differences in levels (e.g., up to two-fold) [40]. While global increases in POA utilization have been rationalized with urgent needs to improve health care, especially for chronic pain, it remains unclear whether Canada is home to higher levels of pain or addresses pain more effectively than comparable nations with substantially lower POA utilization levels; this question continues to be preeminent given the mixed evidence on the efficacy of POAs in the treatment of pain [2,41-44].

Notable changes in POA dispensing trends in Canada, however, occurred between 2011 and 2012, when overall POA – and specifically ‘strong opioid’ – dispensing suddenly decreased in several provinces. The largest proportion of these reductions (>80%) related to reductions in oxycodone dispensing – the POA formulations that had constituted the largest share of ‘strong opioid’ dispensing to date and has been associated with a disproportionate amount of POA-related harm in most provinces [4,18,19]. Reductions in oxycodone dispensing (24% in 2011–2012) were largest in Ontario – the province with the previously highest oxycodone utilization levels in Canada – where total POA dispensing in 2012 was consequently reversed to below-2005 levels [18]. These trend-reversing developments in POA dispensing occurred following – and are likely related to – a variety of recent interventions aiming at improved POA use regulation and control, most prominently including the ‘delisting’ of Oxycontin® (effective March 2012) from the majority of provincial drug formularies [23-25]. Notably, reductions in oxycodone dispensing were not observed in several of the jurisdictions where Oxycontin® was also delisted, yet equally occurred in a province (Alberta) where delisting did not occur. Also given that the present study period captured only a small timeframe (<1 year) following the delisting of Oxycontin®, it remains to be evaluated what further trends and possible changes in POA utilization may follow the above interventions, yet also what factors may be behind the inter-provincially varying trends in oxycodone use from 2011 onward.

While a large proportion of recent policy and media attention regarding POA use and harm in Canada has focused on Oxycontin® and its delisting, this perspective may be unduly narrow and limited, as several other factors could have contributed to the reductions in ‘strong opioid’ dispensing. For example, the extensive media attention on POA related harm, the new National Opioid Guidelines, or widely publicized coroners’ investigations into PO-related overprescribing and deaths (e.g., in Ontario) could have all resulted in more cautious or restrained POA prescribing by physicians [12,21,45]. While none of the above cited events technically forced reductions in POA prescribing, these could have entailed an overall ‘chilling effect’ described in other contexts, making physicians more hesitant or unwilling to prescribe POAs [46-48]. Dasgupta et al. examined the levels and potential impact of media reporting on POA abuse and found a positive correlation [45]; however, in the distinct context of the present study, the impact of media reporting may have contributed to lesser levels of POA prescribing.

Importantly, however, we also observed increases in select other ‘strong opioids’ – specifically, fentanyl and hydromorphone – in most provinces occurring in parallel to the decreases in oxycodone dispensing. These developments could point to a possible (partial) ‘substitution effect’, i.e. that other ‘strong opioids’ were increasingly prescribed where oxycodone utilization has been reduced, as possible development raised as a concern when the broad-based Oxycontin® delisting occurred [24,25,49]. ‘Substitution effects’ have been described for both POAs and non-POA psychotropic medications subsequent to the implementation of tighter regulatory controls or monitoring, entailing shifts in both utilization and harm (e.g., in morbidity or mortality) [50-52].

As a limitation of these analyses, community (i.e., retail pharmacy) dispensing accounts only for a part (yet the large majority) of POA dispensing in Canada; in addition, dispensing amounts do not necessarily equate consumption data yet are a best available and closest measurable proxy indicator of POA consumption.

## Conclusions

In sum, both the key drivers behind, yet especially various key consequences of the observed recent changes in POA dispensing in Canada, need to be systematically evaluated from both clinical and population health perspectives, also given the strong evidence that POA dispensing levels are closely associated with levels of key harm (e.g., non-medical prescription opioid use (NMPOU) morbidity, mortality) indicators [14-16,53]. For example, recent data have found a significant reduction in non-medical POA use in the general Ontario adult population, which may be related to recent reductions in overall POA availability [54]. At the same time, we note the observed key changes in POA dispensing levels occurred prior to the launch of a Canadian ‘National Prescription Drug Use Strategy’ – a package of recommendations and measures aimed at the prevention, surveillance, treatment and enforcement of POA-related problems assembled by various governmental agencies and non-governmental stakeholders – in 2013 [55]. While the implementation and potential effects of these proposed measures remain to be assessed, they could not have had any impact on the data presented here, as the study period ended before these interventions were announced or implemented.

## Abbreviations

AB: Alberta; BC: British Columbia; CA: Canada; CCSA: Canadian Centre on Substance Abuse; DDD: Defined Daily Doses; EMEs: Established market economies; IMSB: IMS Brogan; MN: Manitoba; NB: New Brunswick; NL: Newfoundland; NMPOU: Non-medical prescription opioid use; NS: Nova Scotia; ON: Ontario; PE: Prince Edward Island; POA: Prescription opioid analgesic; QC: Quebec; SK: Saskatchewan; WHO’s ATC: World Health Organization’s Anatomical Therapeutic Chemical.

## Competing interests

The authors declare that they have no competing interests.

## Authors’ contributions

BF and JR designed the study; WJ carried out the statistical analyses. All authors interpreted the results. BF led the writing, to which all authors actively contributed. All authors read and approved the final manuscript.

## Authors’ information

BF, PhD, is Professor and CIHR/PHAC Chair in Applied Public Health in the Faculty of Health Sciences and the School of Criminology, as well as Director of the Centre for Applied Research in Mental Health and Addictions (CARMHA) at Simon Fraser University, Vancouver; he is also a Senior Scientist in Social and Epidemiological Research, Centre for Addiction and Mental Health (CAMH), Toronto, and Professor (status) in the Department of Psychiatry, University of Toronto. His main research foci include the epidemiology and harms of and interventions for illicit and prescription drug misuse.

WJ, MSc, is a Research Associate and Adjunct Professor at CARMHA, Faculty of Health Sciences, Simon Fraser University. He has also worked with the Hospital Planning and Development Department of the Greater Vancouver Regional Hospital District and the University of British Columbia. He has served as a design and statistical analysis consultant to numerous research projects in the mental health and substance use fields; his current research activities include pharmaco-epidemiological analyses of treatment patterns of mental and substance abuse disorders in the general population of British Columbia, Canada.

JR, PhD, is Professor and Inaugural Chair, Addiction Policy, Dalla Lana School of Public Health, University of Toronto, as well as Director, Social and Epidemiological Research (SER) Department, Centre for Addiction and Mental Health (CAMH), Toronto; he is also the Head of the Epidemiological Research Unit, Clinical Psychology and Psychotherapy, at the Dresden University of Technology in Dresden, Germany. His main research foci include global alcohol and prescription opioid use and harm epidemiology; burden of disease and evaluation of interventions and policy for substance use.

## Pre-publication history

The pre-publication history for this paper can be accessed here:

http://www.biomedcentral.com/1472-6963/14/90/prepub
